# stormTB: a web-based simulator of a murine minimal-PBPK model for anti-tuberculosis treatments

**DOI:** 10.3389/fphar.2024.1462193

**Published:** 2025-01-08

**Authors:** Roberto Visintainer, Anna Fochesato, Daniele Boaretti, Stefano Giampiccolo, Shayne Watson, Micha Levi, Federico Reali, Luca Marchetti

**Affiliations:** ^1^ Fondazione The Microsoft Research - University of Trento Centre for Computational and Systems Biology (COSBI), Rovereto, Italy; ^2^ Department of Mathematics, University of Trento, Trento, Italy; ^3^ Department of Information Engineering and Computer Science (DISI), University of Trento, Trento, Italy; ^4^ Gates Medical Research Institute, Cambridge, MA, United States; ^5^ Department of Cellular, Computational and Integrative Biology (CIBIO), University of Trento, Trento, Italy

**Keywords:** physiologically based modeling, pharmacokinetics, pharmacodynamics, ADME, tuberculosis, web interface

## Abstract

**Introduction:**

Tuberculosis (TB) poses a significant threat to global health, with millions of new infections and approximately one million deaths annually. Various modeling efforts have emerged, offering tailored data-driven and physiologically-based solutions for novel and historical compounds. However, this diverse modeling panorama may lack consistency, limiting result comparability. Drug-specific models are often tied to commercial software and developed on various platforms and languages, potentially hindering access and complicating the comparison of different compounds.

**Methods:**

This work introduces stormTB: SimulaTOr of a muRine Minimal-pbpk model for anti-TB drugs. It is a web-based interface for our minimal physiologically based pharmacokinetic (mPBPK) platform, designed to simulate custom treatment scenarios for tuberculosis in murine models. The app facilitates visual comparisons of pharmacokinetic profiles, aiding in assessing drug-dose combinations.

**Results:**

The mPBPK model, supporting 11 anti-TB drugs, offers a unified perspective, overcoming the potential inconsistencies arising from diverse modeling efforts. The app, publicly accessible, provides a user-friendly environment for researchers to conduct what-if analyses and contribute to collective TB eradication efforts. The tool generates comprehensive visualizations of drug concentration profiles and pharmacokinetic/pharmacodynamic indices for TB-relevant tissues, empowering researchers in the quest for more effective TB treatments. stormTB is freely available at the link: https://apps.cosbi.eu/stormTB.

## Introduction

Tuberculosis (TB) poses a significant threat to global health, with millions of new infections and approximately 1.3 million deaths annually (World Health Organization, 2023). Recent initiatives have spurred research in the field, with several novel drug candidates rekindling a pipeline that was nearly empty a decade ago (Dartois and Rubin, 2022). In this context, computational models play a crucial role by rapidly providing information on the exposure and efficacy of new compounds, expediting the drug development process, and aiding in prioritizing the most promising candidates (Dartois and Rubin, 2022).

Alongside this renewed momentum in TB research, various modeling efforts have emerged, offering tailored solutions for both novel and historical compounds. These models suggest dosing strategies and elucidate the effectiveness of monotherapies and drug combinations, *i.e.*, regimens. Data-driven solutions, such as pharmacokinetic (PK) and pharmacokinetic/pharmacodynamic (PK/PD) models, provide effective means to derive exposure and efficacy indexes for TB compounds (Alffenaar et al., 2022; Ernest et al., 2023; Wicha et al., 2018; Zhang et al., 2020). Simultaneously, physiologically based approaches, including physiologically based pharmacokinetic (PBPK) and semi- or fully mechanistic models, provide insights into the intricate diffusion of anti-TB compounds within TB lesions, revealing scenarios that may differ among drugs (Ernest et al., 2021; Humphries et al., 2021; Mehta et al., 2023; Muliaditan and Della Pasqua, 2022).

While the rich and varied landscape of modeling efforts presents valuable insights, it sometimes faces challenges in maintaining uniformity, which can affect the comparability of results. The creation of drug-specific models frequently relies on proprietary software and unfolds across diverse platforms and programming languages, potentially hindering access and complicating the comparison of different compounds. A recent approach is to rely on web interfaces that generate, tune, and simulate (PB)PK models for a wide range of applications. Some examples include igPBPK, an R-based Shiny app for simulating drug withdrawal intervals in cattle or swine for flunixin, florfenicol, and penicillin G with a PBPK model (Chou et al., 2022). ModVizPop is another web app to simulate PK/PD dynamics for compartmental modeling (Vaddady and Kandala, 2021). E-campsis and gPKPDviz are freemium R Shiny apps developed by Calvagone and Genentech, respectively, that allow simulating the PK/PD dynamics with a collection of PK models (Lu et al., 2024; Luyckx, 2024) and the custom integration of thresholds and the area under the curve (AUC) to be displayed in the plots. Still, there is a need for an open-source unified tool specific to anti-TB PBPK drug analysis.

Here, we present a web-based tool tailored for anti-TB drug dynamics and PK/PD metrics in the treatment scenarios to support model-informed treatment development under a unified perspective.

It leverages a previously published minimal physiologically based pharmacokinetic (mPBPK) model that supports 11 historical and under-development anti-TB drugs in pre-clinical murine model commonly used for developing effective anti-TB drugs (Dartois et al., 2024; Reali et al., 2024). Murine models provide critical insights into dosing, tissue distribution, drug stability, probability of relapse, and resistance occurrence. Animal and PK models enable researchers to predict patient drug exposure, optimizing tuberculosis drug regimens. We have consolidated the results from model calibration and variability quantification in the herein introduced R-based web app, stormTB, streamlining model inspection and enabling users to conduct independent what-if analyses using the mPBPK platform. Users can compose a treatment scenario by selecting one drug from the 11 originally included in Reali et al. (2024), the dosage and the treatment length. Scientists can iteratively adjust the experimental settings based on the simulated PK/PD performance and save the results in the workspace. Up to four precomputed monotherapy scenarios can be selected from the workspace and visualized in combination for comparative analysis.

In addition to mean PK profiles, an option for generating a simulation ensemble (SE) for the variability quantification is available, offering suitable choices for population size and the coefficient of variation governing the sampling of clearance and absorption rate values in the population. The tool produces comprehensive visualizations of the drug concentration profile in all nine compartments comprising the mPBPK model, along with descriptions of the PK/PD indices for TB-relevant tissues.

stormTB is freely available at the link: https://apps.cosbi.eu/stormTB.

## Methods

### Minimal-PBPK model

stormTB implements the minimal physiologically based pharmacokinetics model (mPBPK) presented by (Reali et al., 2024) that describes the disposition of 11 anti-pulmonary-TB drugs in murine models. The supported drugs are rifampicin (RIF, R), rifapentine (RPT, P), pyrazinamide (PZA, Z), ethambutol (EMB, E), isoniazid (INH, H), moxifloxacin (MOX, M), delamanid (DEL), pretomanid (PRE, Pa), bedaquiline (BDQ, B), Quabodepistat (QBS, OPC-167832), and GSK2556286 (G286).

The mPBPK model consists of nine ordinary differential equations obtained by streamlining a whole-body mPBPK model via the identification of the tissues least involved in the TB site of action, and the combination of relative compartments to obtain a smaller set of equations (Nestorov et al., 1998; Ryu et al., 2022). Out of the 25 model parameters, only the absorption rate (Ka) and the total clearance (CL) were calibrated using mouse data for each of the 11 drugs and are presented in the app description. A complete list of model parameters is available in Reali et al. (2024).

We present an example of comparison considering the drugs constituting the BPaMZ regimen (Cevik et al., 2024; Xu et al., 2019) at the human equivalent doses: 25 mg/kg of bedaquiline (B), 25 mg/kg of pretomanid (Pa), 100 mg/kg of moxifloxacin (M), and 150 mg/kg of pyrazinamide (Z). The human equivalent dose is set by default when the user selects a drug (Supplementary Table S1) and can be adjusted. In this example, we simulate the drug absorption, distribution, and elimination phases in the first 4 days of daily oral dosing of each compound.

### Implementation and simulations

The original model (Reali et al., 2024), implemented in MATLAB, has been translated into R (4.3.1) and C (gcc 11.4.0) to reduce simulation time and executed using the deSolve (1.40) package (Soetaert et al., 2010). The stormTB user interface is developed with Shiny (1.7.5.1), shinyBS (0.61.1), shinyhelper (0.3.2), shinycssloaders (1.0.0), shinyWidgets (0.8.0), shinyjs (2.1.0), dplyr (1.1.3), tidyr (1.3.0), collapse (2.0.13), zip (2.3.0), ggplot2 (3.4.4), scales (1.2.1), ggiraph (0.8.7) (Attali, 2021; Bailey, 2022; Chang et al., 2024; Csárdi et al., 2023; Gohel and Skintzos, 2023; Krantz, 2024; Mason-Thom, 2019; Perrier et al., 2024; Sali and Attali, 2020; Wickham, 2016; Wickham et al., 2023a; Wickham et al., 2023b; Wickham et al., 2023c).

Each simulation can be computed singularly, applying the reference clearance and absorption rates, or on a simulations ensemble (SE) computing the variability quantification with perturbated parameters. In the latter case, the user can specify the ensemble size (default 100) to be computed, along with the coefficients of variation of the sampled model parameters (CV clearance and CV absorption, both defaulting to 0.3) (Lyons et al., 2013). These values are applied via a lognormal distribution to the two parameters of the specified drug. With the variability quantification option activated, all the statistics are presented with their median and uncertainty expressed as 5th to 95th percentile interval of the values. The same percentiles are represented in all the plots.

To generate the dynamics of the simulation ensemble, stormTB particularly benefits from the translation of the model in C, being able to simulate a test case with a set of a 1,000-parameter perturbations (RIF, 15 days, and default parameters) in 4.3 s (Supplementary Table S2). This is in clear contrast to the 20 min required using a pure R implementation, resulting in a computational time decrease of more than 287-fold. Moreover, for fast postprocessing, we implemented data transformation and basic statistical computation using the R library collapse (written in C/C++). This solution reduced to just 9 s the time needed to compute the test case from the start of the simulation to the rendering of the resulting plots and tables.

### Options

stormTB provides a platform to simulate custom anti-pulmonary-TB treatment scenarios in which the user can choose between the 11 supported drugs and can select the dose amount expressed in mg/kg and number of doses to be administered. When a specific drug is selected from the dropdown menu, the dose is automatically set to its human equivalent as a reference value, the complete set of values is shown in Supplementary Table S1. Users can then freely adjust this value as needed. The number of doses also sets the duration of the simulation assuming the most common setup for anti-TB treatments, which is one oral administration per day. Once the simulation is completed, the user obtains plots and tables summarizing the pharmacokinetics (PK) and pharmacodynamics (PD) of the defined scenario.

**FIGURE 1:**
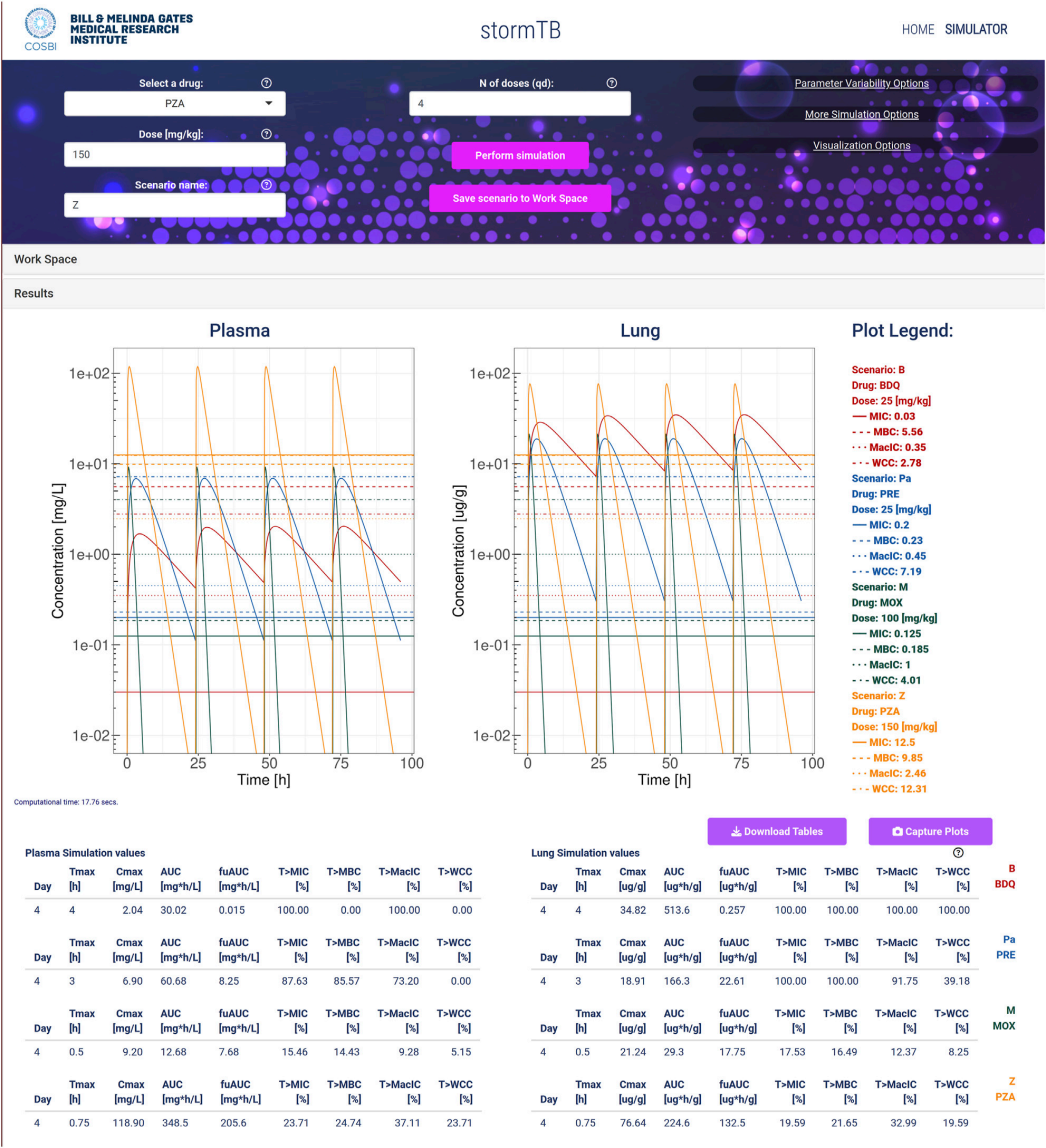
An example of simulation considering the TB drugs involved in the BPaMZ regimen: 25 mg/kg of bedaquiline (B, red), 25 mg/kg of pretomanid (Pa, blue), 100 mg/kg of moxifloxacin (M, green), and 150 mg/kg of pyrazinamide (Z, yellow). The top part of the figure shows the input panel. The central part provides the predicted dynamics of the drug concentrations in plasma (mg/L) and lung (ug/g). Additionally, for each drug, it shows the values for the minimal inhibition concentration (MIC), the minimum bactericidal concentration (MBC), the minimal inhibition concentration in macrophages (MacIC), and the Wayne cidal concentration (WCC). Here, the logarithmic visualization is applied to better appreciate the different dynamics of the drugs, since the Cmax of pyrazinamide is 2 orders of magnitude higher than the others. Note that lung density is assumed 1 g/mL, allowing for direct concentration comparison. The bottom of the figure reports the simulation statistics and refers to the last day of dosing.

**FIGURE 2:**
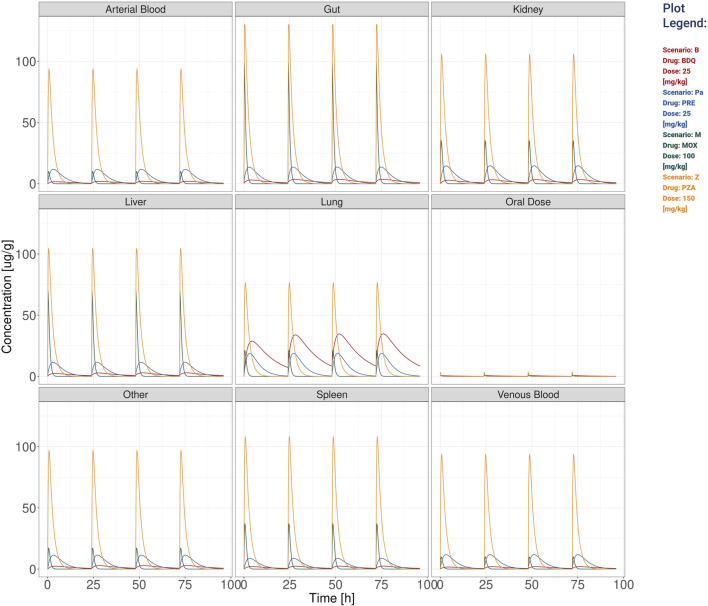
The visualization produced for all the compartments’ simulation considering the TB drugs involved in the BPaMZ regimen: 25 mg/kg of bedaquiline (B), 25 mg/kg of pretomanid (Pa), 100 mg/kg of moxifloxacin (M), and 150 mg/kg of pyrazinamide (Z).

**FIGURE 3:**
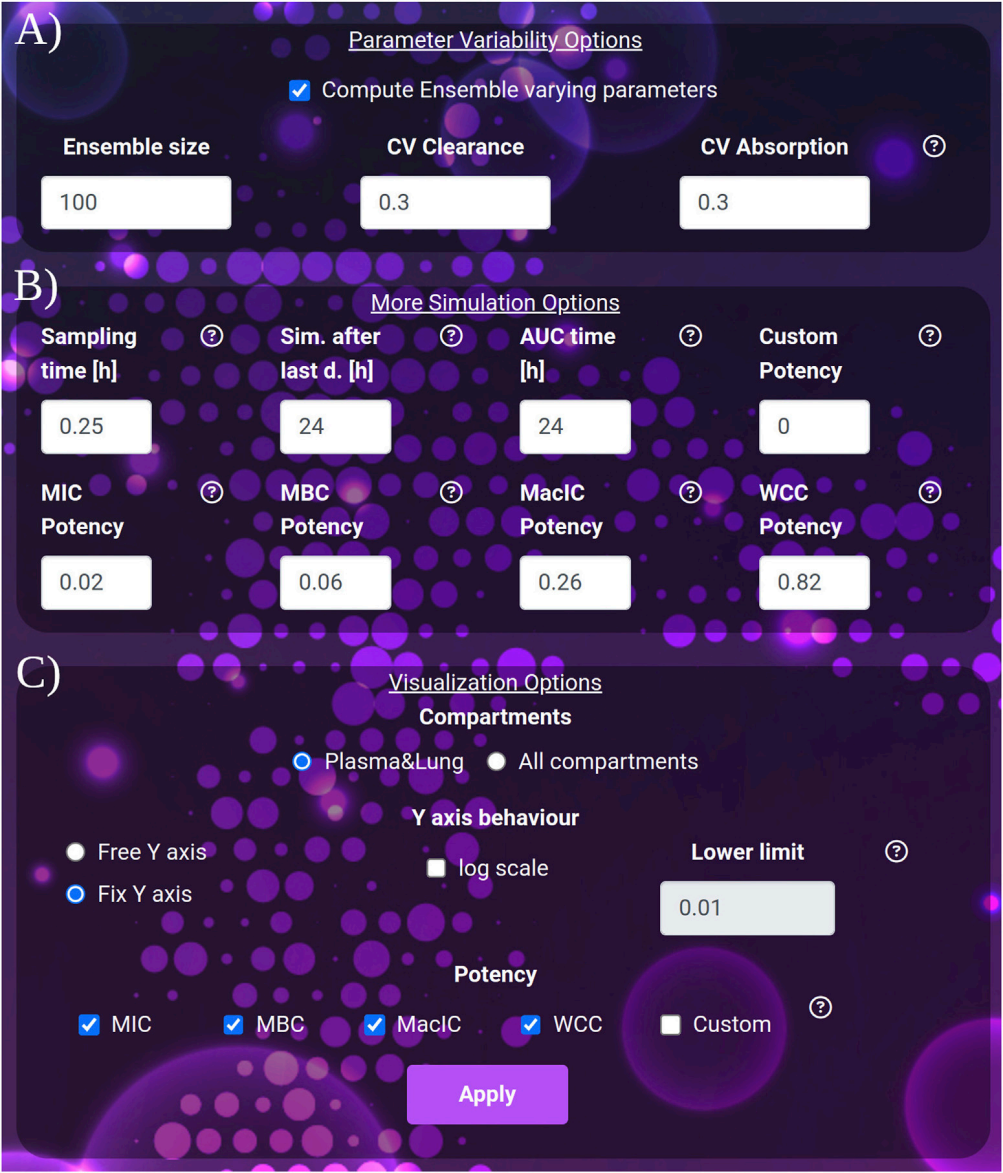
**(A)** The variability quantification options: ensemble size and coefficients of variation to apply to clearance and absorption. **(B)** The extra simulation parameters: the sampling time, the interval to be simulated after the last dose and the interval to be considered for the computation of the AUC after the last dose. **(C)** The visualization parameters: selection between the plasma-lung visualization and all compartments. The *y*-axis behavior: free or fixed scale, application of logarithm and setting of the lower limit of the scale. The potency section: selection of the thresholds to be considered in the plot and result tables.

The default PK plots show the drug concentration profile in plasma and lung compartments, see Figure 1, or the users can analyze PK profiles for all nine model compartments as shown in Figure 2. To better appreciate the dynamics, the visualization can be switched to the logarithmic scale with an adjustable lower limit on the *y*-axis. The user can choose to select just the drugs with comparable dynamics and visualize the plots in a linear scale with a free *y*-axis as shown in Supplementary Figure S1. Here, we exclude from the visualization pyrazinamide, which has a Cmax two orders of magnitude higher than the other drugs, to better appreciate the different behaviors of bedaquiline (B), pretomanid (Pa) and moxifloxacin (M) in all 9 tissues. For plasma and lungs, the plots can include the values of the minimal inhibitory concentration at which at least 50% of the isolates in a test population are inhibited (MIC), the minimal bactericidal concentration at which at least 90% of the isolates are killed (MBC), the concentration that inhibits 90% of growth in macrophages (MacIC) and the Wayne cidal concentration or concentration that kills 90% of dormant bacteria under anaerobic and nutrient rich conditions (WCC) (Lakshminarayana et al., 2015; Wayne and Hayes, 1996). Potency values coming from the literature (Sarathy et al., 2018) or computed by collaborators are automatically set as default thresholds when a drug is selected. Each value can be adjusted by the user to match different susceptibility rates, and in the next simulation, the modified values will be used for statistics and plots. An extra custom threshold with no default value is also available to enrich the set of thresholds that the scientist can explore and visualize. Furthermore, these plots are complemented by tissue-specific tables summarizing important PK and PD indices from the simulation, *i.e.*, the maximum achieved concentration (Cmax), the time at which the maximum concentration is reached (Tmax), the area under the curve for the total amount of drug and for the fraction unbound (AUC, fuAUC), and the time above the potency thresholds (T > MIC50, T > MBC90, T > MacIC90 and T > WCC90). The times above the potency thresholds are computed considering the 24 h after the last simulated dose. The AUC is calculated using the trapezoidal rule on the simulated PK dynamics on the 24 h following the last administration and it can be adjusted between 1 h and the end of the simulation time, see Figure 3.

**FIGURE 4:**
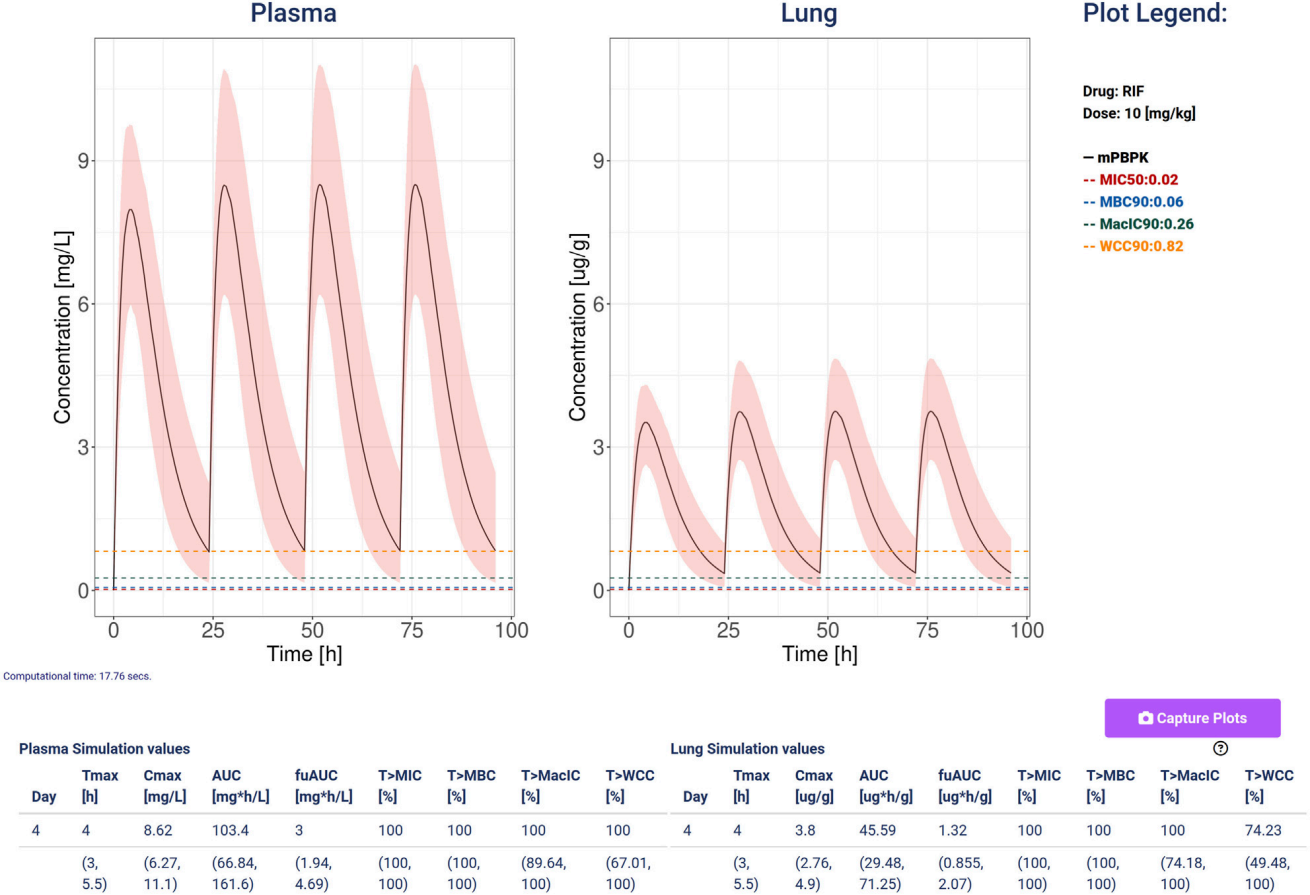
An example of simulation computed with Rifampicin at a dose of 10 mg/kg, 4 doses and a variability quantification ensemble of 100 simulations and coefficient of variation so 0.3 for both clearance and absorption. The plots show the plasma (mg/L) and lung (ug/g) concentrations with the black line representing the median, and the 5–95 percentiles as the shaded pink area. Additionally, it plots and shows the values for the minimal inhibition concentration (MIC, red), the minimum bactericidal concentration (MBC, blue), the minimal inhibition concentration in macrophages (MacIC, green), and the Wayne cidal concentration (WCC, yellow). The tables report the computed statistics showing the last day median and 5–95 percentiles of the simulated ensemble.

For both single mouse and simulation ensembles (Figures 1, 2, 4; Supplementary Figure S1), the web-app offers the option to store and recall simulated scenarios in the workspace area. When recalling saved simulations, the user can visually compare PK profiles of different scenarios, analyzing the impact of various drug-dose combinations with the aid of a combined plot and all the statistics (Figures 1, 2). To guarantee a tidy visualization, the comparison tool supports a maximum of four scenarios of the same length. Plots, tables and parameters of the scenarios can be easily downloaded for reporting purposes, moreover, the simulated data can be saved in raw format for successive analysis with external tools and possibly integrated with results from other software.

Visualization options include

• The simulation Sampling time, expressed in hours (default 0.25, min: 0.1, max: 1), governs the density of simulated points. It affects the post-process time to render the images.
• The duration of the simulation after the last dose, expressed in hours (default and minimum value 24 h, max: 5,000). Increasing this parameter allows the user to exhaustively analyze the dynamics of drugs with very long clearance time such as bedaquiline or rifapentine.
• AUC time, expressed in hours (default 24), indicates the number of hours after the last dose, for which the AUC is computed. AUC time cannot exceed the duration of the simulation after the last dose. Note that AUC time does not apply to the computation of the time above the potency thresholds, which always refers to the 24 h after the last dose.
• For the *Y*-axis, the following options are available: activate the logarithmic scale and set a *Y*-axis lower limit, set the same limit for all the plots shown (Fix *Y*-axis), or automatically adjust to the compartment dynamics (Free *Y*-axis).

## Results

We present stormTB: SimulaTOr of a muRine Minimal-pbpk model for anti-TB drugs, a web-based tool to interact with our minimal PBPK platform to simulate treatment scenarios for tuberculosis in mice. Pharmacokinetics results are reported in terms of AUC, Cmax, and Tmax. In addition, the user is presented with four pharmacodynamics measures expressed as the percentage of the treatment time above each threshold (MIC, MBC, MacIC, and WCC). The use of a customizable simulation ensemble allows the user to enrich the results with percentiles for each quantitative result (Figure 4). To enhance the interoperability of stormTB, we made the raw simulated data available ensuring seamless integration and enhanced flexibility for users to add the results to their projects.

An analysis using our web app typically begins with defining the scenarios under investigation. Each scenario represents a single drug treatment with a dose administered once a day and a specified duration for the simulated experiment, in this case 4 days of dosing. The simulation of a single drug generates PK profile plots for the lung and plasma, and tables display key PK and PD statistics during the final 24 h (day 4). Every simulated monotherapy treatment can be saved to the workspace and be later reloaded singularly or in groups to be compared. The comparative assessment across simulated monotherapy treatments and tissues leads to insights into their effectiveness (Figure 1). Specifically, pyrazinamide is the only component of the BPaMZ regimen less abundant in the lung than in plasma with an AUC ratio of 0.64. It reaches the highest plasma Cmax among the four drugs, however, it shows poor performance in terms of time above MIC and MBC in the lung during the last day of treatment, with 19.59% and 21.65%, respectively. The low level of PZA in lung is confirmed by clinical studies and independent simulations (Mehta et al., 2023; Strydom et al., 2019).

The simulations showed that bedaquiline, pretomanid, and moxifloxacin accumulate more in the lung than in plasma. MIC and MBC thresholds are reached for 100% of the treatment by bedaquiline and pretomanid in the lung, while moxifloxacin, on the last day of treatment, is above the MIC and MBC for 17.53% and 16.49% of the time, respectively. Moreover, bedaquiline and pretomanid performs best in terms of WCC and MacIC, with bedaquiline reaching both thresholds in the lung throughout the whole treatment, followed by pretomanid, with 91.75% and 39.18%, respectively, showing the importance of these two compounds in the BPaMZ regimen to reduce the hard-to-treat bacterial and be effective in TB-lesions (Cevik et al., 2024; Dartois and Rubin, 2022; Reali et al., 2024).

Additionally, stormTB allows to inspect the predicted exposure in all compartments as presented in Figure 2. These allow to quantify the drug penetration in the various compartments, providing predictions that can be instrumental to understand the drug absorption or used for toxicological assessments. To better appreciate the different PK dynamics, the user can select from the workspace drugs with similar Cmax and visualize them in a linear scale (Supplementary Figure S1).

The insights gained from the comparative analysis of the BPaMZ regimen highlight the potential of stormTB in optimizing drug combinations and dosages for enhanced therapeutic efficacy. Such analyses are instrumental for researchers in comparing various drug combinations and doses, designing novel regimens, and fine-tuning dosages to meet effective thresholds.

## Discussion

The mPBPK model simulator stormTB is a versatile, web-based application that streamlines the efforts of both modeling and non-modeling scientists in extracting crucial pharmacokinetic and pharmacodynamic measurements for supported anti-tuberculosis compounds. This simulation tool enables users to access specific PK and PD metrics, analyze compartment-specific PK profiles, and compare them with existing data, thereby promoting efficient benchmarking. While other web interfaces have been available providing a valuable support for researchers in a broad set of applications related to PBPK modeling (Chou et al., 2022; Lu et al., 2024; Luyckx, 2024; Vaddady and Kandala, 2021), we focus on anti-TB drug dynamics and PK/PD metrics in the treatment scenarios.

stormTB enables users to visually compare simulated treatments and assess their pharmacokinetic (PK) and pharmacodynamic (PD) performance—an advanced feature not offered by similar online tools (Figures 1, 2). Our comparison feature is limited to 4 drugs since it is the typical number of drugs co-administered in a regimen and for visualization clarity purposes. While this work focuses on pulmonary TB, the tool simulates and displays PK dynamics across six additional compartments, allowing for evaluation of drug disposition in the liver, spleen, kidneys, gut, arterial, and venous blood (Figure 2). Therefore, this tool embraces the physiologically-derived description of all the compartments that are pivotal in the absorption, distribution, and elimination phases. Users can easily adjust various parameters to fine-tune the simulation, visualization, and computation of a simulation ensemble (SE). The ensemble enables the system to return the median value and 5th–95th percentiles for the generated PK and PD metrics of the simulated plasma and lung concentrations (Figure 3). stormTB is designed with the aim of comparing the effect of different drugs in a simultaneous administration protocol mimicking the routine clinical treatment.

To showcase a possible use of stormTB, we analyzed the BPaMZ regimen, recently tested as possible replacement of the current standard regimen HRZE (Cevik et al., 2024). Focusing on the PK results in lung the concentration of bedaquiline is above all the PD thresholds since the second administration of the treatment and throughout the steady state. MIC50, MBC90 and MacIC90 are met for more than 90% of the treatment by pretomanid; WCC90 – the most stringent threshold–is reached only about the 40% of the time by pretomanid and less that 20% by moxifloxacin and pyrazinamide. Finally, pyrazinamide is the only one that shows an efficacious interval longer in plasma than in lung. The analysis of the four *in vitro* potencies allows a complete investigation of the efficacy of the treatment against replicating and persistent TB strains. The simultaneous treatment of bacteria in different growth conditions, here summarized as reaching the different efficacy thresholds, is crucial to effectively eradicate the infection and reduce the risk of developing pharmaco-resistance.

The webapp suggests that although the results in plasma are in general a good proxy of lung exposure, drugs like bedaquiline, which greatly accumulates in the target tissue, represent exceptions to this rule. In fact, the values of T > MBC90 and T > WCC90 vary from 0 in plasma to 100% in lung, showing the importance of analyzing target attainment at site of action.

stormTB and the original mPBPK on which the app is based have some limitations that we aim at address in future updates (Reali et al., 2024). For example, an updated version of stormTB should integrate enzyme mediated drug metabolism to account for drug-drug interactions (DDI). The refined metabolism description could enable researchers to evaluate the intricate absorption, distribution, metabolism, and excretion (ADME) dynamics of co-administered drugs, thereby shifting the focus towards the complexities of realistic treatment regimens.

Additionally, while the mPBPK model implemented in stormTB already supports different routes of administration, the webapp currently focuses solely on the oral route of administration for consistency. However, in future updates, we aim to provide options for different administration routes, including injectable forms for certain compounds, such as Moxifloxacin, Rifampicin, and Isoniazid. This flexibility will allow for a more comprehensive analysis and tailored treatment planning based on patient needs.

Another important extension of the model would include more animal models, such as rabbits commonly used as efficacy benchmarks in TB, and dog and rat commonly used for toxicological evaluations. Supporting cross-species translations can also lead to early prediction of human exposure and efficacy bridging the gap between preclinical findings and clinical outcomes, potentially informing global health policies and refining TB treatment protocols.

## Conclusion

We present a web application that not only serves as an interface for the model presented in Reali et al. (2024), but can also perform a complete comparative analysis of different therapeutic scenarios producing qualitative and quantitative PK and PD results. The option for users to save output data in a CSV format ensures that users have full access to their data, allowing them to analyze, share, and further process the results with ease, and integrate it with other tools and workflows.

As a user-friendly and freely accessible resource, stormTB democratizes the analysis of a broad spectrum of drugs, both historical and novel, in a unified platform. It empowers TB researchers globally to compare and benchmark drug combinations and dosages, thereby accelerating the discovery and optimization of treatment strategies removing the need for licenses or subscription plans. Through its contributions, stormTB aligns with the collective effort to eradicate TB by the end of the decade, aspiring to make a significant impact on public health.

## Data Availability

Publicly available datasets were analyzed in this study. This data can be found here: https://apps.cosbi.eu/stormTB/.
