# Supplementary material for: stormTB: a web-based simulator of a murine minimal-PBPK model for anti-tuberculosis treatments
Source: Front Pharmacol. 2025 Jan 8;15:1462193. doi: 10.3389/fphar.2024.1462193 (PMC11750688; doi:10.3389/fphar.2024.1462193)
Supplement: Supplementary file 1 [file DataSheet1.pdf]

Supplementary Material - stormTB: A web-based simulator of a murine minimal-PBPK model for anti-tuberculosis treatments.

Roberto Visintainer[1], Anna Fochesato[1,2,+], Daniele Boaretti[1], Stefano Giampiccolo[1,3], Shayne Watson[4], Micha Levi[4], Federico Reali[1,*], and Luca Marchetti[1,5,*]

[1]Fondazione The Microsoft Research – University of Trento Centre for Computational and Systems Biology (COSBI), Italy.

[2]University of Trento, Department of Mathematics, Povo, Italy.

[3]University of Trento, Department of Information Engineering and Computer Science (DISI), Trento Italy.

[4]Gates Medical Research Institute, USA.

[5]University of Trento, Department of Cellular, Computational and Integrative Biology (CIBIO), Trento, Italy.

[+]current affiliation: Roche Pharma Research and Early Development, Roche Innovation Center Basel, Basel, Switzerland.

*** Correspondence:**

Federico Reali reali@cosbi.eu

Luca Marchetti marchetti@cosbi.eu

Figure S1 All compartments PK visualization of: 25 mg/kg of bedaquiline (B), 25 mg/kg of pretomanid (Pa), 100 mg/kg of moxifloxacin (M). Linear scale and free Y-axis visualization is applied; this configuration makes clear the different dynamics of the drugs in the different tissues.

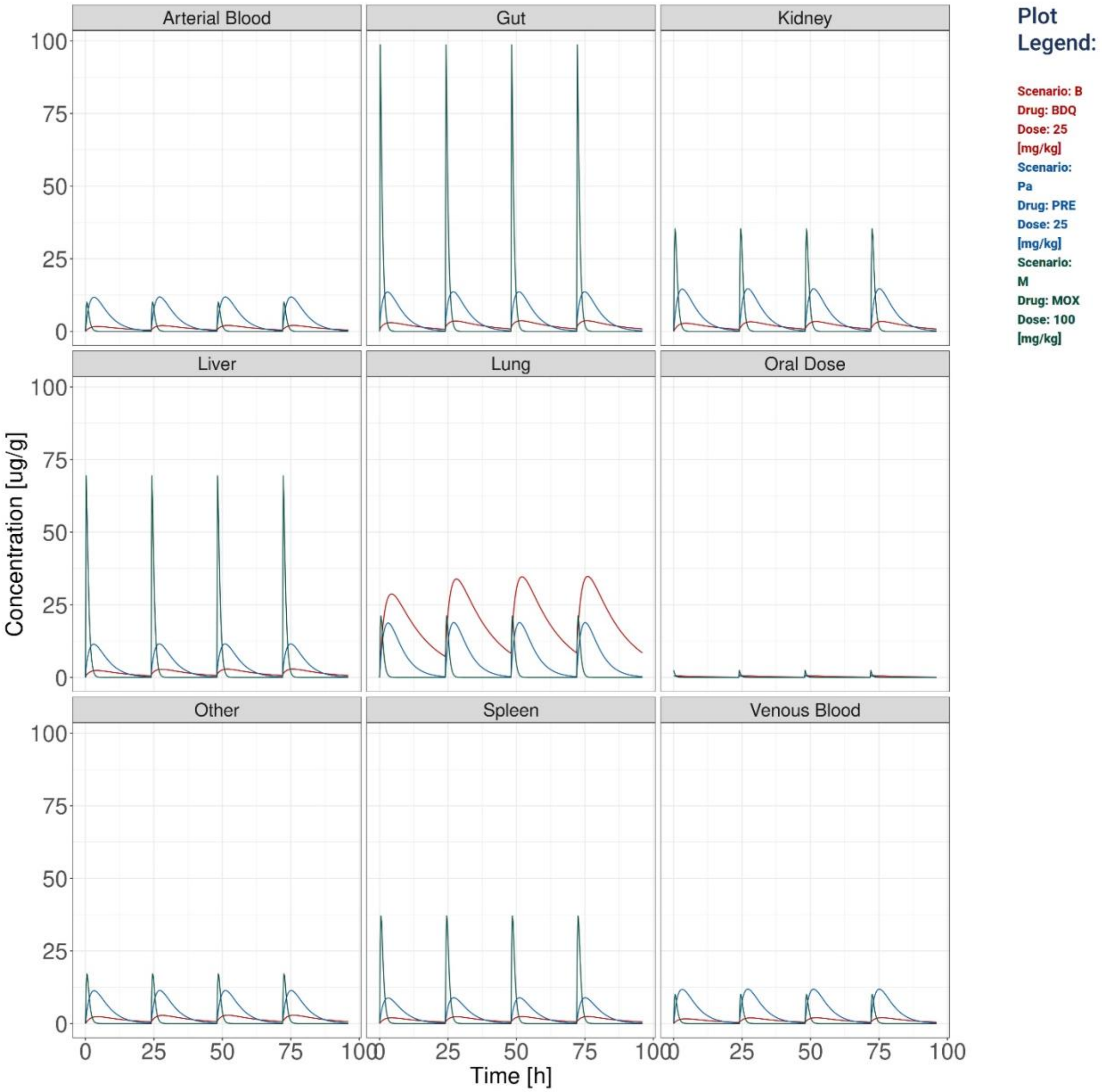

Table S1 Default dose values for each drug corresponding to the human equivalent dose.

Drug	RIF	PZA	DEL	MOX	QBS	G286	EMB	INH	PRE	RPT	BDQ
Dose mg/kg	10	150	3	100	2.5	35	100	25	25	10	25

Table S2 Comparison between pure R and C implementations of computational time for the pure simulation process. We consider 6 different scenarios: number of simulations in the variability simulation ensemble (N SE) [500, 100] and number of doses (N Doses) [5, 10, 15]. We report the median and standard deviation values obtained with 5 repetitions of a simulation of rifampicin and default parameters.

N SE		500			1000		
N Doses		5	10	15	5	10	15
Time (sec) ± SD	R	**195.45** ±2.061	**385.26** ±3.185	**603** ±7.901	**393.54** ±3.751	**801** ±6.125	**1244.4** ±24.8
	C	**1.155** ±0.087	**1.667** ±0.045	**2.23** ±0.062	**2.347** ±0.066	**3.304** ±0.124	**4.335** ±0.129
Fold Change		169.7	231.2	270.4	167.7	242.5	287.1
